# Thrombotic events with or without thrombocytopenia in recipients of adenovirus-based COVID-19 vaccines

**DOI:** 10.3389/fcvm.2022.967926

**Published:** 2022-09-29

**Authors:** Luigi Cari, Mahdieh Naghavi Alhosseini, Alberta Bergamo, Sabrina Pacor, Sabata Pierno, Gianni Sava, Giuseppe Nocentini

**Affiliations:** ^1^Section of Pharmacology, Department of Medicine and Surgery, University of Perugia, Perugia, Italy; ^2^Department of Life Sciences, University of Trieste, Trieste, Italy; ^3^Section of Pharmacology, Department of Pharmacy-Drug Sciences, University of Bari, Bari, Italy

**Keywords:** COVID-19 vaccines, adenovirus-based vaccines, VITT, thrombosis, thrombocytopenia, inflammatory response

## Abstract

COVID-19, the severe acute respiratory syndrome, is one of the major emergencies that have affected health care systems. Drugs and oxygen are only partially effective in saving lives in patients with severe COVID-19, and the most important protection from death is vaccination. The widespread use of COVID-19 adenovirus-based vaccines has provided evidence for the occurrence of rare venous thrombotic events including cerebral venous thrombosis and splanchnic venous thrombosis in recipients of Vaxzevria and Jcovden vaccines and the review focus on them. One year ago, thromboses in Vaxzevria recipients have been associated with thrombocytopenia in the presence of antibodies to platelet factor 4 and have been called vaccine-induced immune thrombotic thrombocytopenia (VITT). The incidence of VITT is equal to 9-31 events per one million doses of vaccines as evaluated by health agencies worldwide and is higher in female and young vaccine recipients. More recently, by using the European EudraVigilance database, it has been demonstrated that the incidence of thrombosis in recipients of adenovirus-based vaccines is 5–10 fold higher than that of VITT and 7–12 fold higher than observed in the recipients of Comirnaty, an mRNA-based vaccine, suggesting that adenovirus-based vaccines cause not only VITT but also thrombosis without thrombocytopenia (non-VITT thrombosis). The incidence of the vaccine-dependent non-VITT thrombosis is different in the adenovirus-based vaccines and the VITT/non-VITT incidence ratio depends on the severity of thrombosis and is inversely related to the age of the recipients. The possible causes and clinical implications of non-VITT thrombosis in vaccine recipients are discussed.

## Efficacy and adverse events of COVID-19 vaccines

### COVID-19 vaccines protect against severe COVID-19

COVID-19, the severe acute respiratory syndrome caused by the SARS-CoV-2 virus, is one of the major emergencies that have affected health care systems and society in recent decades. The clinical signs of COVID-19 depend on the severity of the disease and can be classified as mild, severe, and critical (1). Severe COVID-19 is characterized by oxygen saturation <90% on room air, signs of pneumonia, and trouble breathing. Exacerbation of the disease is defined by hypoxemic respiratory failure, sepsis, septic shock, and impairment of multiple organs, particularly the lungs (pneumonia and acute respiratory distress syndrome), heart (arrhythmias, chest pain), brain (dizziness, headache, impaired consciousness), kidneys, and liver. Many symptoms derive from the inflammatory response to the SARS-CoV-2 virus and the activation of coagulation. The Spike (S) protein may be implicated in the development of both phenomena (2–5).

In patients with a critical disease, drugs and life-sustaining treatments are only partially effective in saving lives (1, 6, 7), and the most important protection from death is represented by vaccination (8–14).

The mRNA-based vaccines Comirnaty (BNT162b2) and Spikevax (mRNA1273) and the adenovirus-based vaccines Vaxzevria (ChadOx1 nCoV-19) and Jcovden (AD26.COV2.S) were the first approved vaccines. Several studies have shown that these vaccines provide important protection against infection, severe COVID-19, and death (11–15). With the advent of omicron, protection against symptomatic disease is relatively poor, ranging from 20 to 80% after the primary vaccine series, but rapidly decreasing over time (0–30% of effectiveness 4–5 months after the last dose) (16–18). Despite the presence of major differences in efficacy following vaccination, as shown by several studies evaluating the same vaccine, it seems reasonable to conclude that protection is slightly lower after vaccination with adenovirus-based vaccines (18). On the contrary, the effectiveness of the vaccines against severe disease and hospitalization is high, ranging from 80 to 100% after the booster dose and remaining constant over time (18).

### Adverse events of COVID-19 vaccines

The adverse events (AEs) observed following vaccination are largely mild or moderate in severity and include fever, chills, headache, flu-like symptoms, muscle pain, and lymphadenopathy (19–22). These events are observed soon after the injection, are not life-threatening and, except for extremely rare anaphylactic reactions, do not require additional treatments. On the other hand, severe AEs (SAEs), although rare, may occur even several days after vaccination and can be life-threatening.

Some SAEs were described for all vaccines, such as myocarditis and pericarditis; in a recently published study (23), the number of these events in young vaccinated males was 11.5, 11.7, and 15.4 events per million doses administered (OMD) of Comirnaty, Spikevax, and Vaxzevria, respectively. These events were reversible and no deaths were observed in under-40 vaccine recipients (23).

Some SAEs occur more frequently with only one/some vaccines, such as Bell’s palsy, caused by Comirnaty and Spikevax (24), and Guillain-Barré syndrome, caused by Jcovden (25). Other SAEs associated with specific COVID-19 vaccines are the venous and arterial thromboses observed in recipients of adenovirus-based vaccines. Our review focuses on these SAEs and their association with thrombocytopenia.

## Venous thrombosis in recipients of adenovirus-based vaccines

### Vaxzevria causes vaccine-induced immune thrombotic thrombocytopenia

The widespread use of COVID-19 vaccines has provided evidence for the occurrence of rare venous thrombotic events in recipients of the Vaxzevria vaccine (26). After two studies demonstrated that, in Vaxzevria recipients, venous thrombotic events [cerebral venous thrombosis (CVT), splanchnic venous thrombosis, and/or other thromboses] are observed in association with platelet aggregation, thrombocytopenia and antibodies to platelet factor 4 (PF4) (27, 28), such event was called vaccine-induced immune thrombotic thrombocytopenia (VITT). VITT is similar to heparin-induced thrombocytopenia (HIT) and is considered an adverse event caused by Vaxzevria (26). Vaccine-induced anti-PF4 antibodies are thought to be responsible for these thrombotic complications because anti-PF4 antibodies were rarely found in CVT patients in the past (i.e., before VITT) (29, 30).

The role of anti-PF4 antibodies in adenovirus-based vaccines was recently confirmed (31, 32). Baker et al. performed a computational simulation showing the electrostatic interaction of adenoviruses with PF4, which was experimentally confirmed by surface plasmon resonance. Greinacher et al. showed that vaccine components can form antigenic complexes with PF4 on platelet surfaces, which become targets of anti-PF4 antibodies in patients with VITT. In addition, they demonstrated that approximately 50% of the protein content of the Vaxzevria vaccine belongs to T-REx-293, the cells in which it is produced, and that EDTA, an excipient of the Vaxzevria vaccine, causes local vascular leakage that favors the systemic dissemination of the vaccine components (32). Finally, a confirmation of the pathogenesis of VITT comes from a washed platelet aggregation-based assay (32).

### Incidence of vaccine-induced immune thrombotic thrombocytopenia in Vaxzevria recipients

After the discovery that Vaxzevria causes VITT, the incidence of VITT was classified as very rare by the European Medicines Agency (EMA) (26) but with the accumulation of data, regulatory agencies stated that in Vaxzevria recipients, 1-3 SAEs per 100,000 vaccinations are VITT. In particular, EMA, the Medicines and Healthcare products Regulatory Agency (MHRA, UK), Health Canada, and the Australian Technical Advisory Group on Immunisation (ATAGI) reported 9.3, 15.1, 21.9, and 27–31 VITT/OMD, respectively (33–36).

By evaluating SAEs from each patient report and considering only patients in whom both thrombosis and thrombocytopenia were reported, we showed that the VITT incidence in the European EudraVigilance database (EEd) was 19.4 SAEs/OMD in Vaxzevria recipients (37). The frequency of VITT reports is about 40 folds greater than that shown in Comirnaty recipients. Similar results were found in South Korea (38) even if the incidence of VITT in Vaxzevria recipients was lower than that observed in EEd.

Study findings and the variable incidence of VITT observed worldwide by regulatory agencies may be due to several factors, including genetics and co-morbidity of vaccinated populations, besides the fact that the sex and age of Vaxzevria recipients may also play a role. For example, we found that the incidence of CVT in Vaxzevria recipients was much higher in young people and women of childbearing age than in adult males and the elderly (39). In particular, a sixfold difference in CVT incidence was found between 18 and 24 years and 60–69 years Vaxzevria recipients (39). Thus, the difference in the incidence of VITT after the Vaxzevria vaccine in South Korea compared with Europe may be due, at least in part, to the age of vaccinee. In fact, in South Korea only 18.3% of the vaccinated people were under 50 years of age, whereas in Europe, these were 37.1% of the population (38, 39).

### The incidence of thrombosis after Vaxzevria is much higher than that of vaccine-induced immune thrombotic thrombocytopenia

In Vaxzevria recipients, the incidence of thrombosis including CVTs, splanchnic vein thromboses, thromboembolic diseases, and “other” venous thrombotic events (with or without thrombocytopenia) was about 5 fold more (110.6 SAEs/OMD) than the above-reported incidence of VITT (37). Moreover, in the same recipients, the incidence of thromboses and thrombosis-related deaths was much higher than that observed in Comirnaty recipients, used as a baseline reference (37). Two more studies demonstrated an increased risk of venous thrombosis in Vaxzevria than in Comirnaty and Spikevax recipients (40, 41).

Given clinicians’ knowledge of VITT and the recommendation of regulatory authorities to check platelet count in the presence of thrombosis (42), underreporting of low platelet count is unlikely, and data suggest that most thromboses observed in Vaxzevria recipients, despite being caused by vaccination, occur without thrombocytopenia (non-VITT thrombosis). Non-VITT thrombosis following Vaxzevria vaccination has been reported by case studies and shows a persistently normal platelet count and can occur both in the presence and in the absence of anti-PF4 antibodies (43–46).

### In Vaxzevria recipients, the VITT/non-VITT thrombosis incidence ratio is severity-dependent and inversely correlated with age

In Vaxzevria recipients, relevant differences in the association between thrombosis and thrombocytopenia are observed depending on the severity of thrombosis. Indeed, thrombocytopenia is associated with 18% of thrombotic SAEs and 48% of thrombosis-related deaths (37). Therefore, the non-VITT/VITT thrombosis incidence ratio is equal to 4.6 in thrombotic SAEs and 1.1 in thrombosis-related deaths. Moreover, thrombocytopenia is associated with 49% of CVT, one of the most severe thrombosis, and 77% of CVT-related deaths (37).

**FIGURE 1:**
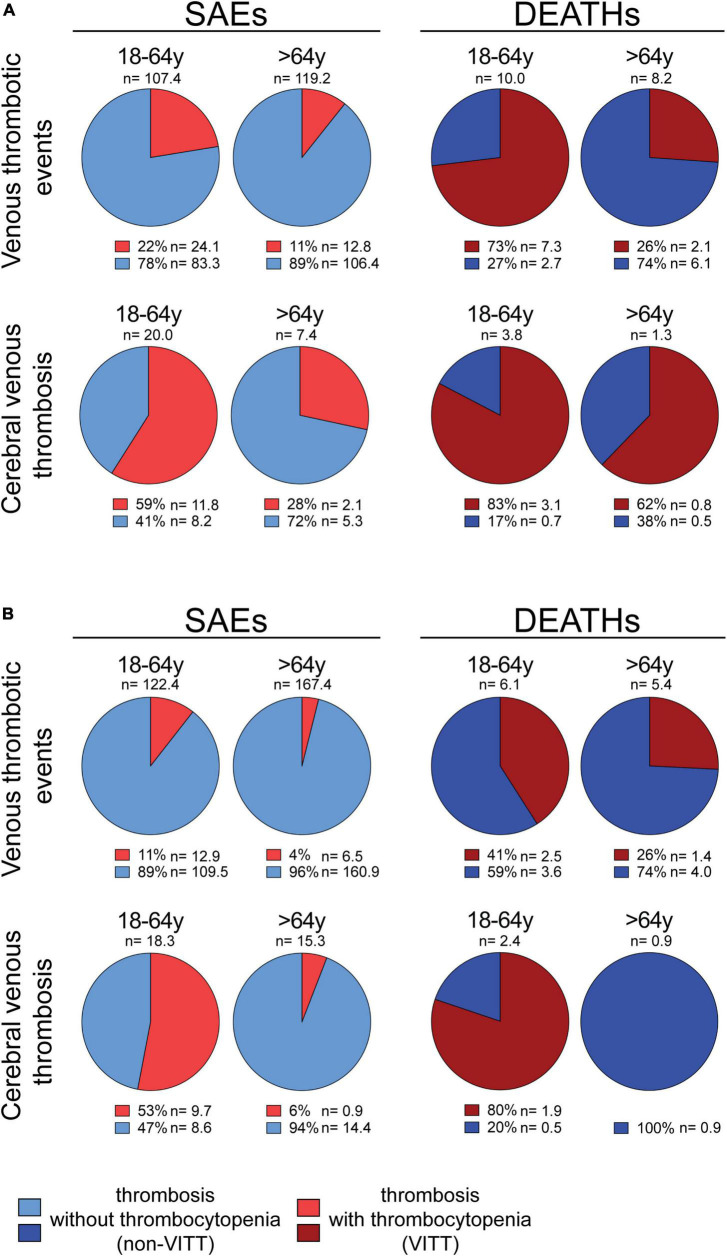
Percentage of thrombotic events associated or not with thrombocytopenia in Vaxzevria and Jcovden recipients. **(A, left panel)** All venous thrombotic events (cerebral venous thromboses, splanchnic vein thromboses, thromboembolic diseases, and “other” venous thrombotic events) or only cerebral venous thromboses, reported as SAEs in the EudraVigilance database following the Vaxzevria vaccine are shown. The number of events/OMD (n) and the percentage of individual SAEs associated (light red) or not (light blue) with thrombocytopenia are reported [data from (37)]. **(A, right panel)** The deaths due to all venous thrombotic events (cerebral venous thromboses, splanchnic vein thromboses, thromboembolic diseases, and “other” venous thrombotic events) or cerebral venous thromboses in the EudraVigilance database following the Vaxzevria vaccine are shown. The number of events/OMD (n) and the percentage of deaths associated (dark red) or not (dark blue) with thrombocytopenia are reported [data from (37)]. **(B, left panel)** All venous thrombotic events (cerebral venous thromboses, splanchnic vein thromboses, thromboembolic diseases, and “other” venous thrombotic events) or only cerebral venous thromboses, reported as SAEs in the EudraVigilance database following the Jcovden vaccine are shown. The number of events/OMD (n) and the percentage of individual SAEs associated (light red) or not (light blue) with thrombocytopenia are reported [data from (37)]. **(B, right panel)** The deaths due to all venous thrombotic events (cerebral venous thromboses, splanchnic vein thromboses, thromboembolic diseases, and “other” venous thrombotic events) or only cerebral venous thrombosis in the EudraVigilance database following the Jcovden vaccine are shown. The number of events/OMD (n) and the percentage of deaths associated (dark red) or not (dark blue) with thrombocytopenia are reported [data from (37)].

In addition, the incidence of the thrombocytopenia/CVT association is even higher in young-adults representing 83% of Vaxzevria recipients who died because of CVT (37) (Figure 1A). Data are consistent with the results published by Greinacher et al. that considered deadly Vaxzevria-dependent CVT to be almost always associated with thrombocytopenia (27). Therefore, thrombocytopenia is more frequent in life-threatening thromboses than in non-fatal ones and in young-adults than in the elderly.

### Incidence of vaccine-induced immune thrombotic thrombocytopenia in Jcovden recipients

Cerebral venous thrombosis (CVT) has also been observed in Jcovden recipients (47, 48), making VITT one of the severe AEs of Jcovden (49). Notably, the incidence of VITT in Jcovden recipients is lower than that observed in Vaxzevria recipients (37). The lower incidence of VITT in Jcovden as compared to Vaxzevria recipients is observed both in young-adults (12.9 vs. 24.1 SAEs/OMD) and in the elderly (6.5 vs. 12.7 SAEs/OMD) (37). Even VITT-related deaths are less frequent in Jcovden as compared to Vaxzevria recipients (37).

### The incidence of thrombosis after Jcovden is much higher than that of vaccine-induced immune thrombotic thrombocytopenia

As in the case of Vaxzevria, the incidence of Jcovden-dependent thrombosis is much higher (131 SAEs/OMD) than the incidence of VITT (11.2 SAEs/OMD) (37), suggesting that most vaccine-dependent thromboses are unrelated to thrombocytopenia. The incidence of VITT decreases with the age of the vaccinee (37). Indeed, thrombocytopenia is associated with 11% and 4% of thrombotic SAEs in young-adults and elderly, respectively (Figure 1B) (39) and, in those who die, thrombocytopenia is associated with about 41 and 26% of the thrombotic SAEs in young-adults and elderly, respectively (Figure 1B) (39). Moreover, the incidence of VITT increases with the severity of SAEs (Figure 1B) (37). An exception to the trend of VITT incidence is the virtual absence of VITT in the elderly with CVT or dying of CVT (Figure 1B) (37). One case report described the occurrence of non-VITT thrombosis in Jcovden recipients (50).

### Differences between Vaxzevria- and Jcovden-dependent thrombosis

The VITT/non-VITT incidence ratio in Vaxzevria and Jcovden recipients is different, although both are adenovirus-based vaccines. While Vaxzevria recipients show a twice higher incidence of VITT than Jcovden recipients, the incidence of non-VITT thrombosis is higher in Jcovden recipients than in Vaxzevria recipients (120 and 91 SAEs/OMD, respectively) (37). In addition, the incidence of thrombosis and thromboembolic diseases is similar in Vaxzevria and Jcovden young-adult recipients and is higher in Jcovden than Vaxzevria elderly recipients (37).

## Arterial thrombosis in recipients of adenovirus-based vaccines

**FIGURE 2:**
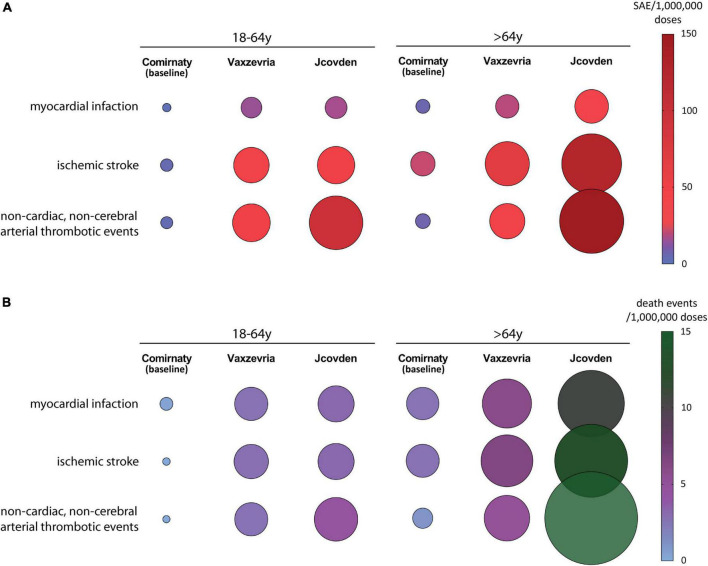
The incidence of arterial thrombotic events in the EudraVigilance database following Vaxzevria and Jcovden vaccines are shown. The incidence of events following Comirnaty is reported for comparison. **(A)** Incidence of individual SAEs/OMD related to arterial thrombotic events in young-adult **(left panel)** and elderly **(right panel)** vaccine recipients [data from (37)]. **(B)** Incidence of death events/OMD related to arterial thrombotic events in young-adult **(left panel)** and elderly **(right panel)** vaccine recipients [data from (37)].

The incidence of arterial thrombotic events [myocardial infarction, ischemic stroke, and non-cardiac, non-cerebral arterial thrombotic events (37)] in Vaxzevria and Jcovden recipients has been compared with that in Comirnaty, used as baseline control (37). The incidence of myocardial infarction reported in EEd in young-adult recipients of adenovirus-based vaccines is about sixfold higher, compared with Comirnaty, and an even higher incidence was observed in other arterial SAEs (Figure 2A), suggesting that Vaxzevria and Jcovden vaccines not only cause venous thrombosis events but also cause arterial thrombosis. Specifically, in Jcovden elderly recipients, the excess of ischemic stroke over Comirnaty is 106 SAEs/OMD, causing an excess of 10.5 deaths/OMD (37). A recent study, confirms that Vaxzevria and Jcovden vaccines are associated with myocardial infarction (relative incidence, 1.29 and 1.75, respectively), even if significance concerning the SAE due to Jcovden was not reached, probably due to the low number of vaccinations with Jcovden (50).

Regarding arterial SAEs, Jcovden causes more SAEs and SAE-dependent deaths than Vaxzevria (Figure 2B).

## Discussion

### Are VITT and non-VITT thromboses caused by different mechanisms?

It can be hypothesized that non-VITT thrombosis can occur in two ways. First, thrombosis is caused by anti-PF4 antibodies that activate platelets only at specific sites and do not cause thrombocytopenia [thrombosis with anti-PF4 antibodies without thrombocytopenia syndrome (T4-noTS)]. T4-noTS would mean that platelets, activated by low concentrations of anti-PF4 antibodies, aggregate mainly at sites where predisposing factors (e.g., pro-inflammatory environment, blood stasis) are present without causing massive consumption of platelets and thrombocytopenia. According to this hypothesis, T4-noTS would be caused by the same mechanisms leading to VITT. The hypothesis seems to be confirmed by the higher incidence of thrombocytopenia in vaccine recipients who die due to thrombosis as compared to those who survive.

The second way to envision non-VITT thrombosis is to hypothesize that anti-PF4 antibodies are not involved when thrombosis is not associated with thrombocytopenia [thrombosis without anti-PF4 antibodies without thrombocytopenia syndrome (T-no4TS)]. T-no4TS could be caused by factors promoting platelet aggregation, such as an inflammatory response favored by adenovirus-based vaccines and local predisposing factors. The hypothesis seems to be confirmed by the higher incidence of arterial thrombotic events in adenovirus-based vaccine recipients, the different incidence of thrombosis and VITT between Vaxzevria and Jcovden recipients, and the different ages at which VITT and non-VITT thrombosis are observed.

### How can vaccines cause pro-thrombotic effects independent of anti-PF4 antibodies?

Azzarone et al. hypothesized that in vaccine recipients suffering from local inflammatory reaction, an early event is represented by the production of interleukin 6 (IL-6) (3) which favors endothelial cells reactivity and induces plasminogen activator inhibitor-1 (51, 52), the main physiological inhibitor of the plasminogen activators in the bloodstream (53). A confirmation of the hypothesis came from an observational study demonstrating that 24-48h following Vaxzevria vaccination, the IL-6 levels were almost doubled (54).

Interestingly, another observational study demonstrated that in healthy young-adults vaccinated with Vaxzevria, hemostatic changes were still present after about one month from vaccination (55). In particular, the levels of Von Willebrand Factor (VWF) and active VWF, indicating endothelial activation, were increased 39% and 24%, respectively. Moreover, faster thrombin generation (i.e., more active coagulation system) and lower coagulations levels (use of coagulation factors) were observed, suggesting that the hemostatic system of vaccinee is shifted to a more procoagulant state compared to unvaccinated controls. Therefore, non-VITT thrombo-embolic events may be the clinical expression of procoagulant coagulation profile, consequent to a pro-inflammatory trigger, represented by vaccination with adenovirus-based vaccines.

### How do vaccine components determine pro-inflammatory/pro-thrombotic events?

In search of potential pro-inflammatory and pro-thrombotic triggering factors present in virus-based vaccines, we found three factors: the S protein (2, 4, 56, 57), the adenovirus particles (3, 31), and the cellular debris present in vaccine preparation (32).

The presence of S protein in blood vessels and tissues other than the muscle into which the vaccine is injected can be hypothesized. The S protein may induce pro-inflammatory cytokines and chemokines in macrophages (56, 57) and T cells (58), directly damage endothelium (4), activate platelets (5), and autoimmune response, the latter triggered by antigenic epitopes of S protein shared with molecular chaperones (59). However, given the lower incidence of thrombosis and arterial SAEs in Comirnaty recipients, S protein appears to be not sufficient to cause such events, at least at the concentration determined by the Comirnaty vaccine.

Viral blood dissemination was detected either in patients treated with infusions of adenoviral vectors in the tumor or the right hepatic artery (51, 60). Indeed, after the injection of Vaxzevria into mice, sporadic small amounts of the virus were found in other tissues (61). Adenoviral particles seem to not promote platelet aggregation (3), but the receptors mediating the binding of adenoviral particles to cells are expressed by human endothelial cells, platelets, and erythrocytes (3, 31).

Finally, it should also be considered that the Vaxzevria and Jcovden vaccines contain residual cellular proteins from the cell lines by which adenoviruses are produced likely causing platelet activation (32).

If the reasons why adenovirus-based vaccines cause non-VITT thrombosis and arterial events might be those mentioned above, it is currently impossible to say which is the main culprit. Their synergism is likely crucial.

### Why is the incidence of severe adverse events different in recipients of Vaxzevria and Jcovden?

The different incidences of VITT, non-VITT thromboses, and arterial events in recipients of Vaxzevria and Jcovden may be explained by the differences between the two vaccines, which can be summarized as follows. First, the type of adenovirus is different and it has been demonstrated that chimpanzee adenovirus (Vaxzevria) has a stronger negative charge than the human Ad26 virus (Jcovden) (62). Moreover, molecular simulations suggest that the Vaxzevria’s adenovirus charge and shape could allow it to bind to the positively charged PF4 protein (31). Second, the amount of infectious units in the Jcovden vaccine is 3.3-fold higher than that in the Vaxzevria vaccine (49, 63), possibly implying a higher S protein and adenoviral protein burden. Third, the human cell lines by which adenoviruses are produced are different: T-REx-293 cells for Vaxzevria and PER.C6 TetR cells for Jcovden (64). Furthermore, approximately 50% of the protein content of the Vaxzevria vaccine belongs to T-REx-293 (32), while Jcovden seems to be less contaminated by cell line debris (64). Finally, EDTA is present in the Vaxzevria vaccine but not in the Jcovden vaccine (49, 63) and may favor the development of inflammation at the vaccine inoculation site.

## Concluding remarks

The incidence of VITT, non-VITT thrombosis, and arterial events, although rare, is much higher in recipients of Vaxzevria and Jcovden vaccines than in recipients of the Comirnaty vaccine (37, 38), suggesting a causal relationship between these events and the adenovirus-based vaccines. We hypothesize that the venous and arterial thromboses observed with adenovirus-based vaccines and observed in absence of thrombocytopenia are due to the combination of at least three triggering factors, all of which may be involved in vascular inflammation and coagulation, and several arguments suggest that it is independent of anti-PF4 antibodies (T-no4TS). However, further studies are needed to confidently exclude that non-VITT thromboses are due to low levels of anti-PF4 antibodies.

In Vaxzevria recipients with thrombosis, the incidence of VITT is very high when they die due to CVT. However, in the presence of thrombosis other than CVT, the incidence of VITT is lower, and it is further reduced in the elderly and young adults who do not die. Therefore, in recently vaccinated individuals, the possibility that a thrombosis or arterial event is caused by adenovirus-based vaccines should be considered even in the absence of thrombocytopenia, favoring a correct diagnosis and providing important information for the management of treatment.

## Author contributions

GN with the contribution of LC, MNA, AB, SPa, SPi, and GS: conceptualization. GN, LC, and GS: writing—original draft. All authors contributed to the article and approved the submitted version.
